# Exposure to Estrone Impairs Oogenesis and Spawning in Zebrafish (*Danio rerio*)

**DOI:** 10.1007/s00244-026-01202-8

**Published:** 2026-06-17

**Authors:** Yves Moreira Ribeiro, Davidson Peruci Moreira, Alessandro Loureiro Paschoalini, Nilo Bazzoli, Elizete Rizzo

**Affiliations:** 1 Programa de Pós-graduação em Biologia Celular, Departamento de Morfologia, Instituto de Ciências Biológicas, Universidade Federal de Minas Gerais, UFMG, Belo Horizonte, Minas Gerais Brazil; 2 Departamento de Anatomia e Imagem, Faculdade de Medicina, Universidade Federal de Minas Gerais, UFMG, Belo Horizonte, Minas Gerais Brazil; 3 Programa de Pós-graduação em Biodiversidade e Meio Ambiente, Pontifícia Universidade Católica de Minas Gerais, PUC Minas, Belo Horizonte, Minas Gerais Brazil

## Abstract

Estrone (E1) is one of the major natural estrogens influencing the reproductive system of vertebrates. However, it contaminates aquatic environments due to runoff from livestock waste and the discharge of domestic sewage. The aim of this study was to investigate the impact of exposure to environmentally relevant concentrations of E1 on oogenesis and spawning in zebrafish (*Danio rerio*). Fish were exposed, in duplicate, to 20, 200, and 2000 ng/L of E1 for 49 days for oogenesis analysis evaluating histology, sex steroids, morphometry, cell proliferation, apoptosis, immunohistochemistry for insulin-like growth factor (IGF) and inducible nitric oxide synthetase (iNOS). 17β-estradiol (E2) and 11-ketotestosterone (11-KT) were assessed by ELISA assay. Nitric oxide production was evaluated indirectly by quantifying nitrite. Results revealed that E1 exposure altered the proportions of ovarian follicles and somatic components, with significant increase of oogonia, follicular atresia and inflammatory infiltrate, and decrease of follicular cells. Levels of E2, and immunoreaction for IGF1 increased in E120 and 200 ng/L groups, but 11-KT, IGF2 and IGF1R were not altered. Nitrite concentrations in ovaries were significantly elevated in E1 200 and 2000 ng/L groups, but iNOS immunoreaction was not altered. These changes led to reduced egg production in all groups exposed to E1 and significatively decreased fertilization rates at 200 and 2000 ng/L. Overall, the findings demonstrate that E1, even at concentrations commonly found in surface waters, has detrimental effects on ovarian development, gametogenesis and reproductive success in zebrafish. Monitoring environmental estrogens levels in aquatic environments is therefore essential for fish conservation.

## Introduction

Exposure to environmental estrogens disrupts the reproductive activity of vertebrates by altering gonadal gene expression, sex steroid production, and ultimately reducing or eliminating the ability to generate offspring (Adeel et al. 2017; Nadal et al. 2018). Once absorbed, estrogens bind to estrogen alpha and beta receptors (ERα/β) or G-protein-coupled estrogen receptors (GPER) (Vo et al. 2019; Pillerová et al. 2021). These binding trigger cell signaling pathways which modify the synthesis, metabolism, and excretion of various hormones (Kabir et al. 2015; La Merrill et al. 2020), thus being considered endocrine disruptors chemical (EDCs). Since estrogen receptors are expressed in various vertebrate tissues and organs (Sánchez-Criado et al. 2004; Frank et al. 2014), EDCs can compromise several metabolic and physiological processes, with reproduction being particularly affected (Nelson and Habibi 2023; Adeel et al. 2017). Studies have demonstrated that exposure to natural and synthetic estrogens impairs gametogenesis and fish reproduction, leading to reduced fecundity and fertility, altered sexual differentiation, and disrupted sex steroid production (Jackson and Klerks 2019; Rutherford et al. 2020; Lei et al. 2023).

In the fish ovary, gametogenesis begins with the mitotic division of oogonia, with the potential for self-renewal and differentiation into new oocytes, which then initiate meiosis (Quagio-Grassiotto et al. 2011; Elkouby and Mullins 2017). During meiosis, oocytes arrest at the diplotene of prophase I, and subsequently individualize, together with surrounding granulosa cells, to form the ovarian follicle. As the follicle develops, specialized theca and follicular (granulosa in mammals) cells produce 17β-estradiol, which stimulates the liver to synthesize vitellogenin and choriogenin—precursor proteins of the yolk and zona radiata in fish eggs (Lubzens et al. 2010, 2017). In general, oogenesis, follicular growth, oocyte maturation, and spawning are regulated by pituitary-derived gonadotropins and sex steroids (Chu et al. 2015; Borella et al. 2020).

In addition to hormones, insulin-like growth factors (IGFs) play crucial roles in vertebrate reproduction, regulating cell proliferation, differentiation, and survival during gametogenesis through paracrine and autocrine mechanisms (Reinecke 2010; Breves et al. 2018; Neirijnck et al. 2019). In female fish, IGFs are expressed in follicular cells, theca cells, and oocytes, with both IGF1 and IGF2 being involved in oocyte maturation and capable of triggering the resumption of meiosis and germinal vesicle breakdown (GVBD), depending on the species (Reinecke 2010). In rainbow trout, IGFs may have synergistic actions with the maturation-inducing steroid (MIS) but cannot induce resumption of meiosis directly and are not potent inducers of oocyte maturation competence (Weber 2023).

Along with hormones and growth factors, other signaling molecules such as nitric oxide (NO) participate in gametogenesis and oocyte maturation. NO is produced by nitric oxide synthase (NOS), and its synthesis can be stimulated by estrogens in various tissues (Nevzati et al. 2015; Choudhury and Saha 2016). Three NOS isoforms—endothelial NOS (eNOS), inducible (iNOS) and neuronal NOS (nNOS)—were detected in the active ovary of *Clarias batrachus*, predominantly expressed in the thecal and granulosa cells of the growing and fully-grown follicles (Singh and Lal 2015, 2017). In ovary, NO contributes to angiogenesis, steroidogenesis, and redox regulation, acting as an important modulator of gonadal physiology (Athanasiou et al. 2025). Despite its physiological functions, elevated levels of NO-derived compounds can be detrimental, causing DNA damage and oxidation of proteins and lipids (Mikkelsen and Wardman 2003; Forstermann and Sessa 2012). The harmful effects of NO are primarily mediated by peroxynitrite (ONOO^−^), a highly reactive compound formed by the reaction of NO with superoxide anion (O₂·⁻) (Luo et al. 2021).

Environmental estrogens, including estrone (E1), 17β-estradiol (E2), 17α-ethinylestradiol (EE2) and estriol (E3) enter aquatic ecosystems through livestock runoff and wastewater effluents, posing threats to fish reproduction, population sustainability, animal health, and human well-being (Andaluri et al. 2012; Adeel et al. 2017; Silva et al. 2025). Although E1 is less potent than other estrogens, it is a significant endocrine disruptor because it often occurs at higher concentrations than other natural estrogens (E2 and E3) in surface waters across several countries (Grzegorzek et al. 2024). E1 is commonly detected in surface waters worldwide, with concentrations ranging from 10.17 to 256.66 ng/L in South America, up to 28.8 ng/L in China, and up to 4.433 ng/L in Europe (Wang et al. 2015; Weber et al. 2017; Glineur et al. 2024).

Despite the impacts of E1 on human health and aquatic ecosystem, few studies have investigated its effects on fish gametogenesis and spawning. This knowledge gap is particularly concerning because regulatory agencies in North and South America have not yet established maximum allowable concentrations for E1 in continental waters (USEPA 2002; CONAMA 2005), while its ecological effects remain insufficiently understood. In this context, zebrafish are a well-established experimental model for studies of reproductive toxicology and endocrine disruption (Schäfers et al. 2007; Moreira et al. 2023; Lima et al. 2026). Previous studies have demonstrated that exposure of male zebrafish to E1 induces a marked reduction in 11-KT levels, leading to decreased sperm production and impaired semen quality (Ribeiro et al. 2023). However, the effects of E1 on female reproductive processes remain largely unknown. Given this gap, we hypothesized that chronic exposure to E1 impairs both oogenesis and spawning in zebrafish. Accordingly, the present study aimed to evaluate the impacts of environmentally relevant concentrations of E1 on these reproductive endpoints.

## Materials and Methods

In this study, the animals were handled according to the Guide for Animal Experimentation established by the Brazilian College for Animal Experimentation (COBEA) and the project was approved by the Ethics and Animal Use Committee of the Federal University of Minas Gerais (protocol CEUA 115/2020).

### Experimental Design

For this study, 120-day-old wild-type zebrafish (*Danio rerio*) were obtained from a local supplier. The fish were acclimated for 15 days in two 120 L tanks at 26–28 °C under a 12L:12D photoperiod, with biological filtration, and continuous aeration. They were fed twice a day with a commercial diet (Tetramin flakes, Germany). Then, the fish were sexed based on external morphological characteristics and transferred to ten 25 L aquariums (*n* = 10 fish per aquarium) in a 1:1 male to female ratio. After acclimation, zebrafish were exposed for 49 days to E1 to encompass multiple spawning cycles, allowing evaluation of consistent effects of E1 exposure on ovarian dynamics and spawning. The experiment included five treatment groups, which were performed in duplicate: control (water only), ethanol control (0,001% ethanol), E1 at 20 ng/L, E1 at 200 ng/L and E1 at 2000 ng/L. The concentrations of 20 and 200 ng/L of E1 are environmentally relevant (Wang et al. 2015; Weber et al. 2017; Glineur et al. 2024), whereas the concentration of 2000 ng/L is artificially high. A stock solution of E1 (1 mg/mL; Sigma-Aldrich) was initially prepared in absolute ethanol and subsequently added to the aquaria to achieve the target concentrations (20, 200, and 2000 ng/L), with ethanol standardized to 0.001% across all treatments. During the exposure period, biological filters were removed, and 80% of the water was renewed every two days to maintain safe levels of ammonia, pH and dissolved oxygen. Given the half-life of 3 to 4 days for E1 in the water (Adeel et al. 2017), a new dose of E1 was added whenever the water was replaced. Water changes were also performed in the control and ethanol groups to ensure consistency across all treatments. To avoid cross-contamination, freshly prepared water containing the respective treatment concentrations (E1 at 20, 200 and 2000 ng/L) was added separately to each aquarium during water renewal. In addition, water handling was performed carefully to avoid splashing or transfer of exposure water between aquaria.

Throughout the exposure period, water parameters (Table 1) were monitored weekly using thermometers and commercial kits (LabconTest, Brazil). To ensure that the nominal concentrations of E1 were maintained within the expected, water samples were collected after the first and third week of exposure to quantify E1 concentrations by high-performance liquid chromatography coupled with mass spectrometry (HPLC–MS) as previously standardized (Weber et al. 2017; Table 1). For this, a Shimadzu Prominence HPLC SPD-20A UV–Vis liquid chromatograph equipped with a Kromasil C18 column (250 mm, 4.6 mm, 2 µm particle size) and a Bruker Autoflex III MALDI-TOF/TOF mass spectrometer were used. The samples were analyzed in duplicate, and the detection and quantification limits were 1.71 ng/L and 2.34 ng/L, respectively. The physicochemical parameters of the water (Table 1) remained within the optimal ranges for zebrafish cultivation: temperatures ranging from 27 to 28.5 °C, pH from 6.0 to 7.0, dissolved oxygen levels from 6.0 to 7.2 mg/L, and ammonia concentrations below 0.01 mg/L. E1 was undetectable in control and ethanol groups. In the treated groups, concentrations of E1 were 19.78, 229.10 and 1941.92 ng/L for the E1 20, 200 and 2000 ng/L groups, respectively (Table 1).

**Table 1:** Physicochemical parameters and estrone (E1) concentrations in the water

Treatment	Temperature (°C)	pH	Oxygen (mg/l)	Ammonia (mg/l)	E1 (ng/L)
Control	28.18 ± 0.22	6.53 ± 0.08	6.50 ± 0.14	0.006 ± 0.001	< LQ
EtOH	28.46 ± 0.28	6.80 ± 0.13	6.71 ± 0.16	0.007 ± 0.001	< LQ
E1 20 ng/L	27.93 ± 0.39	6.36 ± 0.06	6.29 ± 0.55	0.007 ± 0.001	19.78 ± 1.63
E1 200 ng/L	28.18 ± 0.26	6.53 ± 0.10	7.07 ± 0.20	0.006 ± 0.001	229.10 ± 7.31
E1 2000 ng/L	28.36 ± 0.13	6.50 ± 0.08	6.79 ± 0.21	0.006 ± 0.001	1941.92 ± 2.81

Values are expressed as mean ± SEM, < LQ = < quantification limit

At the end of the exposure period, fish were euthanized, and biometric measurements were collected from each female, including total length (TL), body weight (BW) and gonadal weight (GW). The gonadosomatic index (GSI = 100 GW/BW) and Fulton condition factor (K = 100 BW/TL^3^) were subsequently calculated to assess reproductive and overall health status of each fish.

### Histology and Morphometry

For the analysis of gametogenesis, ovarian samples (*n* = 6 specimens per group) were collected immediately after euthanasia and fixed in Bouin’s solution for 8 h at room temperature as previously reported by Weber et al (2019). After fixation, the tissues were washed, dehydrated through a graded ethanol series, 70%, 80%, 90%, 100%, and 100%, cleared in three consecutive baths of fresh xylene, embedded in paraffin, sectioned at 5 µm thickness, and stained with hematoxylin–eosin. For morphometric evaluation, five randomly chosen cross-sections from each specimen (totaling 30 fields per group) were examined at 200 × magnification using the Zeiss Axio Vision E64 software coupled to a light microscope. To quantify the proportion (%) of the ovarian follicle types (perinucleolar, cortical alveoli, vitellogenic, postovulatory, and atretic follicles), digital images were analyzed using ImageJ software, using a 540-points graticule according to Sales et. al. (2025). Other components (fibrosus components, blood vessels, and inflammatory infiltrates) were also quantified. Oogonia and follicular cells were counted at 400 × magnification. The proportion (%) was calculated as the number of points over a given structure divided by the total number of graticule points, multiplied by 100. Additionally, the diameters of 150 largest vitellogenic follicles were measured per group.

### Immunohistochemistry and TUNEL Assay

Six ovarian samples per group were fixed in 4% paraformaldehyde in 0.1 M phosphate buffer pH 7.3 for 12 h at room temperature (Thomé et al. 2012). After fixation, the tissues were processed as previously described above. For immunohistochemistry, sections were incubated with 3% H_2_O_2_ in phosphate-buffered saline (PBS) for 30 min at room temperature to block endogenous peroxidase activity. Antigen retrieval was performed using 10 mM citrate buffer (pH 6.0) for 20 min at 95 °C. Non-specific binding was blocked with 2% bovine serum albumin in PBS for 30 min at room temperature. Sections were then incubated overnight at 4 °C with the primary antibody (Table 2) in a humid chamber. Detection was carried out using the Dakocytomation LSAB kit (goat secondary antibody conjugated with biotin and streptavidin-peroxidase), followed by staining with 3,3′-diaminobenzidine (DAB; Sigma-Aldrich, USA) and counterstained with hematoxylin. Negative control was prepared by omitting the primary antibody. In the negative control slides, the entire immunohistochemical procedure was repeated with the primary antibody omitted. The immunohistochemical protocols and primary antibody concentrations used in this study were performed according to previous studies (Prado et al. 2019; Moreira et al. 2023).

**Table 2:** Primary antibodies and dilutions used in immunohistochemistry

Primary antibody	Source	Code	Dilution
Monoclonal anti-PCNA	Sigma-aldrich	P8825	1:100
Polyclonal Anti-IGF1	Santa cruz biotechnology	Sc-9013	1:100
Polyclonal Anti-IGF2	Santa cruz biotechnology	Sc-5622	1:100
Polyclonal Anti-IGF1R	Santa cruz biotechnology	SC-713	1:100
Polyclonal Anti-iNOS	Sigma-aldrich	SAB4502012	1:150

Quantification of the immunoreactivity was performed in five randomly chosen cross-sections, totaling 30 fields examined per group at 400 × magnification. For cell proliferation detection in oogonia and follicular cells, proliferating cell nuclear antigen (PCNA) was used, and the immunoreactions were examined using the Zeiss Axio Vision E64 software coupled to a light microscope. For IGF1, IGF2, IGF1R, and iNOS, optical densitometry (OD) of staining was measured using Fiji/ImageJ software (https://imagej.net/Fiji/Downloads) with the Color Deconvolution 1.7 plugin (vector H DAB) as previously reported by Ribeiro et al. (2023). OD was calculated for each image according to the formula OD = log 10 (maximum intensity/(mean intensity-background intensity)) (Ruifrok and Johnston 2001).

For cell death, the sections were analyzed in situ using the TUNEL (terminal deoxynucleotidyl transferase mediated dUTP nick end labeling) assay with the QIA33 kit (TdT-FragEL DNA fragmentation. Calbiochem, USA) as described in previous studies (Ribeiro et al. 2021; Moreira et al. 2023). Briefly, sections were inactivated for endogenous peroxidase, incubated with the terminal enzyme deoxynucleotidyl transferase (TdT) for 1 h at 37 °C and then with anti-digoxigenin conjugated with peroxidase for 30 min. The peroxidase reaction was revealed with DAB, and the sections were counterstained with hematoxylin. For the negative control, one section was not treated with the TdT enzyme.

PCNA and TUNEL-positive cells were quantified in 30 fields per group at 400 × magnification using the Zeiss Axio Vision E64 software coupled to a light microscope. Cell proliferation and death was determined from the percentage of PCNA or TUNEL-positive cells in relation to the total number of these cells.

### ELISA Assay

Ovarian samples (*N* = 6 per group) were stored to − 80 °C for the quantification of 17β-estradiol (E2) and 11-ketotestosterone (11-KT), following previously established protocols (Ribeiro et al. 2021). For this, standardized samples with 150 µg of tissue were homogenized in extraction buffer (50 mM Tris–HCl, pH 8.0, containing 0.002% aprotinin and 1 mM phenylmethylsulfonyl), the extracts were vortexed and centrifuged at 15,000 × g for 60 min at 4 °C. The resulting supernatants were analyzed in duplicate using commercial ELISA kits (Cayman Chemical Company, Michigan, USA), with detection limits of 6.6 pg/ml for E2 and 1.3 pg/ml for 11-KT. Absorbance was measured at 405 nm using a microplate reader (BioTek Instruments. USA).

### Nitric Oxide Quantification

Levels of NO were assessed, in duplicate, indirectly by measuring nitrite (NO_2_^−^) concentrations using the Griess reaction as described by Zheng et al. (2015). Ovarian samples (*N* = 6 per group) were collected and stored at − 80 °C. For analysis, samples were homogenized in extraction buffer (50 mM Tris–HCl, pH 8.0, 0.002% aprotinin and 1 mM phenylmethylsulfonyl), then the extracts were vortexed and centrifuged at 15,000 g for 60 min at 4 °C. A 100 µL aliquot of the supernatant was mixed with 100 µL of Griess reagent (1% sulfanilamide, 0.1% N-(1-naphtyl)ethylenediamine dihydrochloride, and 5% phosphoric acid) and the mixture was incubated in the dark at room temperature for 10 min. Absorbance was measured at 540 nm using a microplate reader (BioTek Instruments, USA). Nitrite concentrations were determined from the absorbance values using a sodium nitrite (NaNO_2_) standard curve.

### Spawning and Fertilization

To assess the effects of the treatments on spawning and fertilization, an egg collection box was placed in each aquarium every seven days and retrieved the following morning for analysis. The total number of eggs released was counted using a stereoscopic microscope. The fertilization rate (%) was then calculated as the proportion of embryos that reached the blastula stage (Kimmel et al. 1995) relative to the total number of eggs produced.

### Statistical Analysis

All statistical analyses and graphs were performed using GraphPad Prism version 6. Data normality was assessed with Shapiro–Wilk test. Because biometric data, biological indices, ovarian morphometry, sex steroid concentrations, cell proliferation, cell death, and immunoreaction reactions did not meet assumptions of normality, these variables were analyzed using the Kruskal–Wallis test followed by Dunn's post-test. Nitrite concentrations were evaluated using one-way ANOVA followed by Tukey's post hoc test. Results for treated groups were compared to the control group (water) and were expressed as mean ± standard error (SEM). Statistical significance was set at *p* < 0.05. No significant difference was observed between the control and ethanol (EtOH) group for any variable analyzed.

## Results

The biological parameters of the fish did not differ significantly among groups, including TL (4.26–4.43 cm), BW (0.92 –1.08 g) and K (1.12 –1.30) between control and treated groups (*p* > 0.05).

### Gonadosomatic Index, Sex Steroids and Vitellogenic Follicle Diameter

**Fig. 1:**
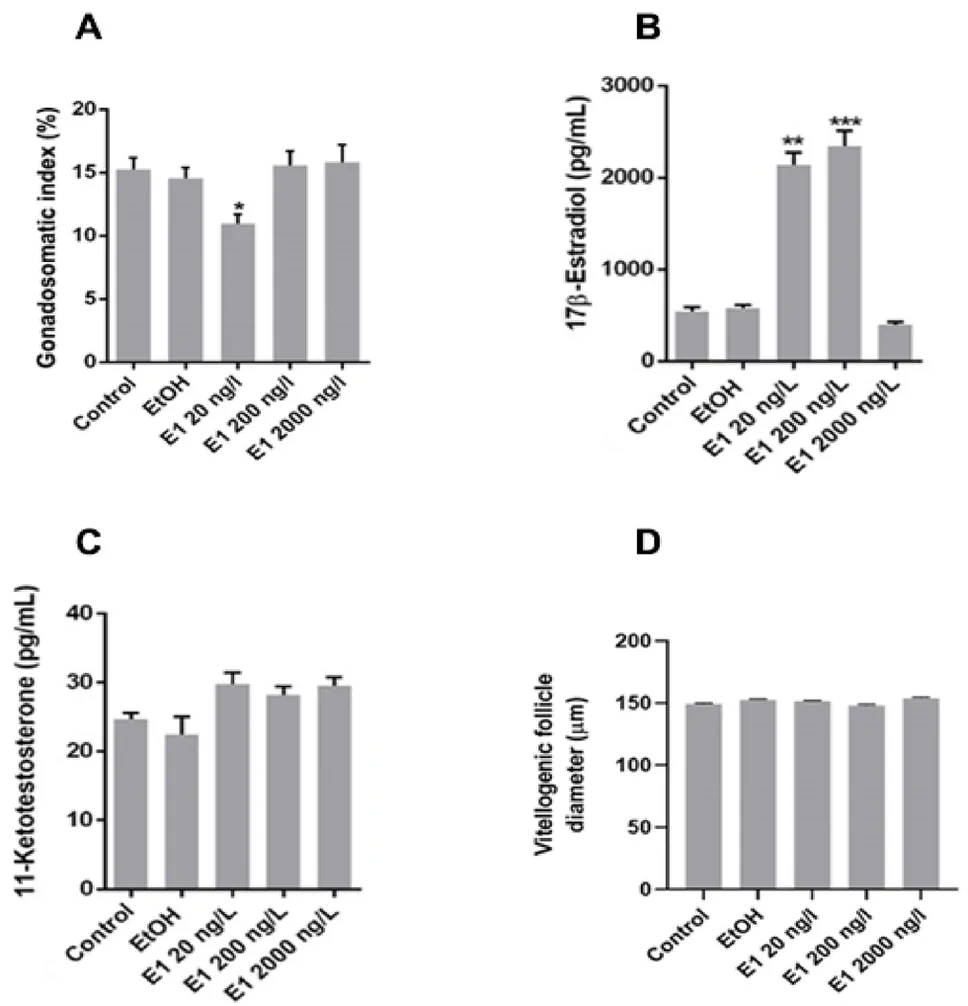
**A** GSI, **B** concentrations of E2 and **C** 11-KT, and **D** vitellogenic follicle diameters in ovaries of *Danio rerio* exposed to 20, 200 and 2000 ng/L of E1 for 49 days. Data are expressed as mean ± SEM from six fish per group (*p < 0.05, **p < 0.01, ***p < 0.001)

The GSI values for the group exposed to 20 ng/L of E1 were significantly lower than those of the control group (*p* < 0.05; Fig. 1A). Additionally, ovarian concentrations of E2 were significantly elevated in the E1 20 ng/L and 200 ng/L groups (*p* < 0.01 and *p* < 0.001, respectively), whereas no significant difference was observed in the E1 2000 ng/L group (*p* > 0.05; Fig. 1B). In contrast, 11-KT did not differ significantly among groups, although a slight upward trend was observed in the E1-exposed groups (Fig. 1C). The diameters of the largest vitellogenic follicles in the treated groups ranged from 121.20 to 172.47 µm, with no significant difference compared to the control group (149.3 ± 0.75 µm, *p* > 0.05; Fig. 1D). However, a slight trend towards increased values was observed in the treated groups (Fig. 1).

### Morphometry of Gametogenesis

**Fig. 2:**
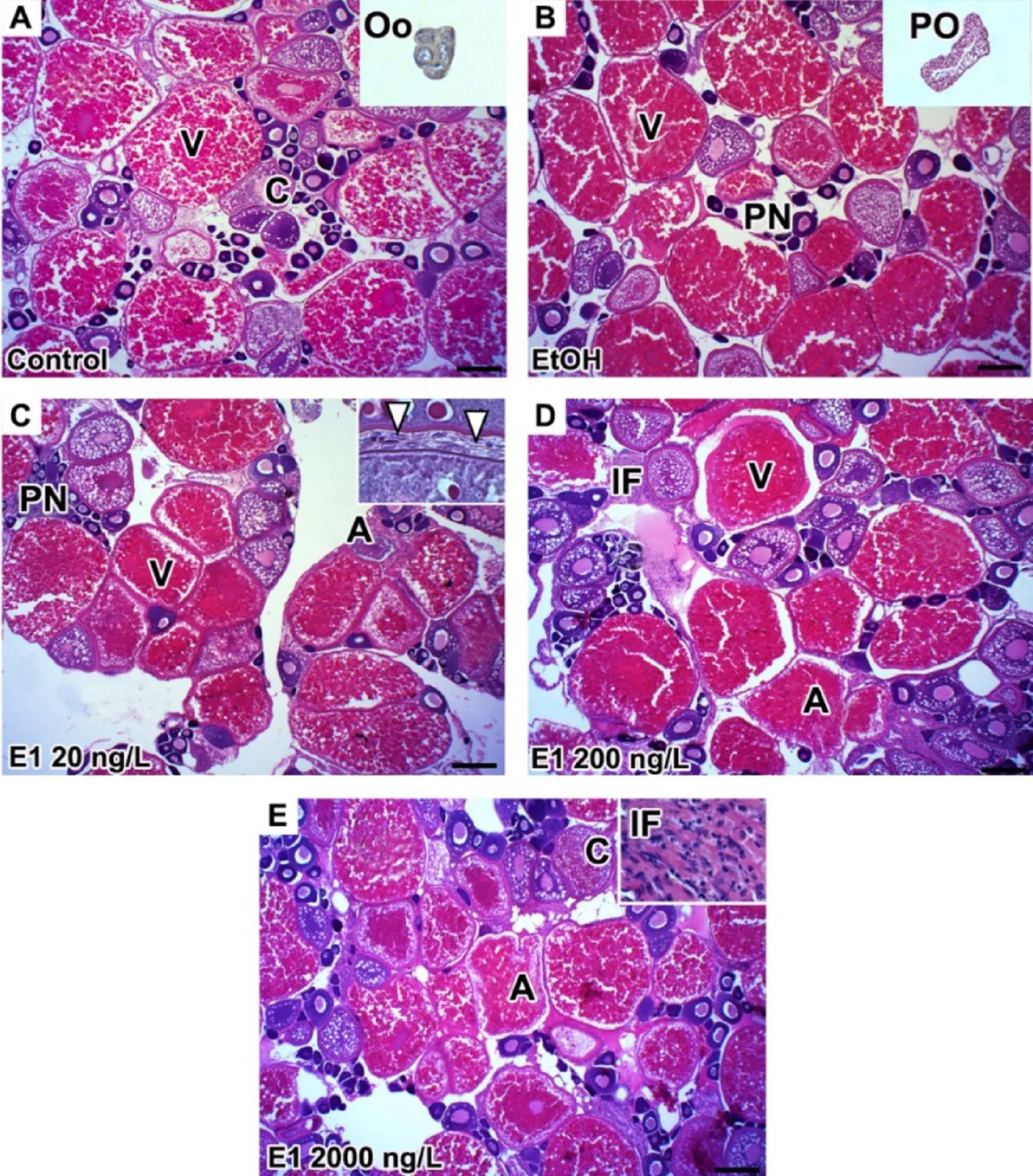
Ovarian histological sections in *D. rerio* exposed to E1 for 49 days. **A** Control, **B** ethanol, **C** E1 at 20 ng/L, **D** E1 at 200 ng/L and **E** E1 at 2000 ng/L. Sections were stained with hematoxylin and eosin. Females from both the controls and E1-treated groups showed maturing ovaries, with oogonia (Oo) and follicles at various stages of development: (PN) perinucleolar, **C** cortical alveoli, (V) vitellogenic, (PO) postovulatory, and (A) atretic follicles. In detail, note oogonia nests (in A), follicular cells (arrowhead in C) and inflammatory infiltrate (IF in E). Bar scale = 50 µm

**Fig. 3:**
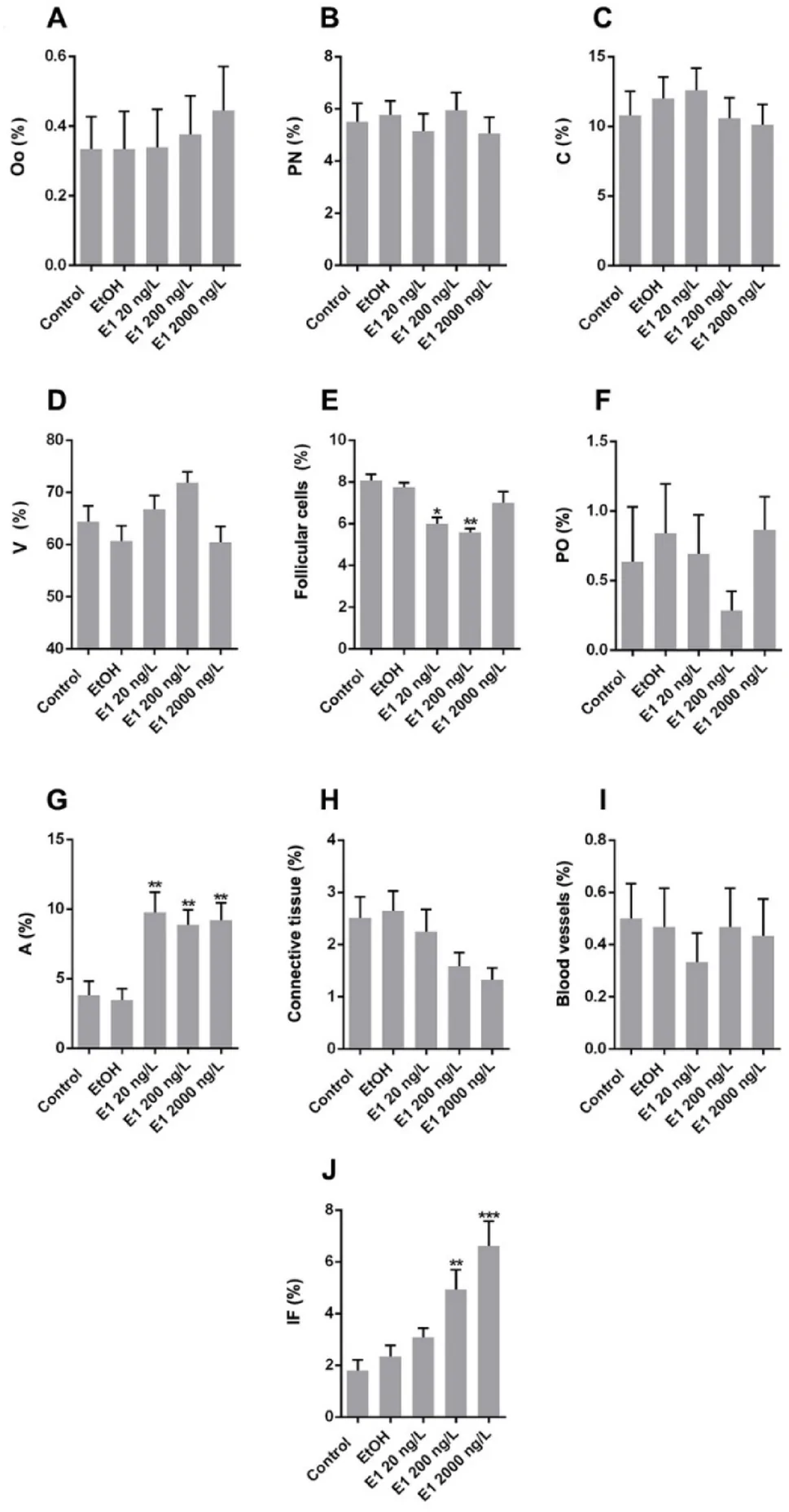
Proportion (%) of oogonia, follicles and somatic components in the ovaries of *D. rerio* exposed to E1 for 49 days: **A** Oogonia (Oo), **B** perinucleolar follicles (PN), **C** cortical alveoli follicles (**C**), **D** vitellogenic follicles (V), **F**, postovulatory follicles (PO), **G** atretic follicles (**A**), (**H**) connective tissue, (**I**) blood vessels, and (**J**) inflammatory infiltrate (IF). Note significantly reduced follicular cells at E1 20 and 200 ng/L groups (**E**) and increased follicular atresia in treated groups (**G**) and inflammatory infiltrate at E1 200 and 2000 ng/L groups (in **J**). Data are expressed as mean ± SEM from six fish per group (*p < 0.05, ** p < 0.01, *** p < 0.001)

Histological analyses revealed that all females, both in the control and E1-exposed groups, exhibited maturing ovaries with follicles at different developmental stages (Fig. 2). Morphometric evaluation indicated no significant differences between the treated and control groups in the proportions of perinucleolar, cortical alveoli, vitellogenic, and postovulatory follicles, as well as connective tissue and blood vessels (Fig. 3B–F). However, the proportion of follicular cells was significantly reduced in the E1 20 ng/L and 200 ng/L groups compared to the control (*p* < 0.05; Fig. 3E). Additionally, the proportions of atretic follicles and inflammatory infiltrate were significantly higher in the E1 200 ng/L and 2000 ng/L groups (*p* < 0.01; Fig. 3G, F).

### Cell Proliferation and Apoptosis

**Fig. 4:**
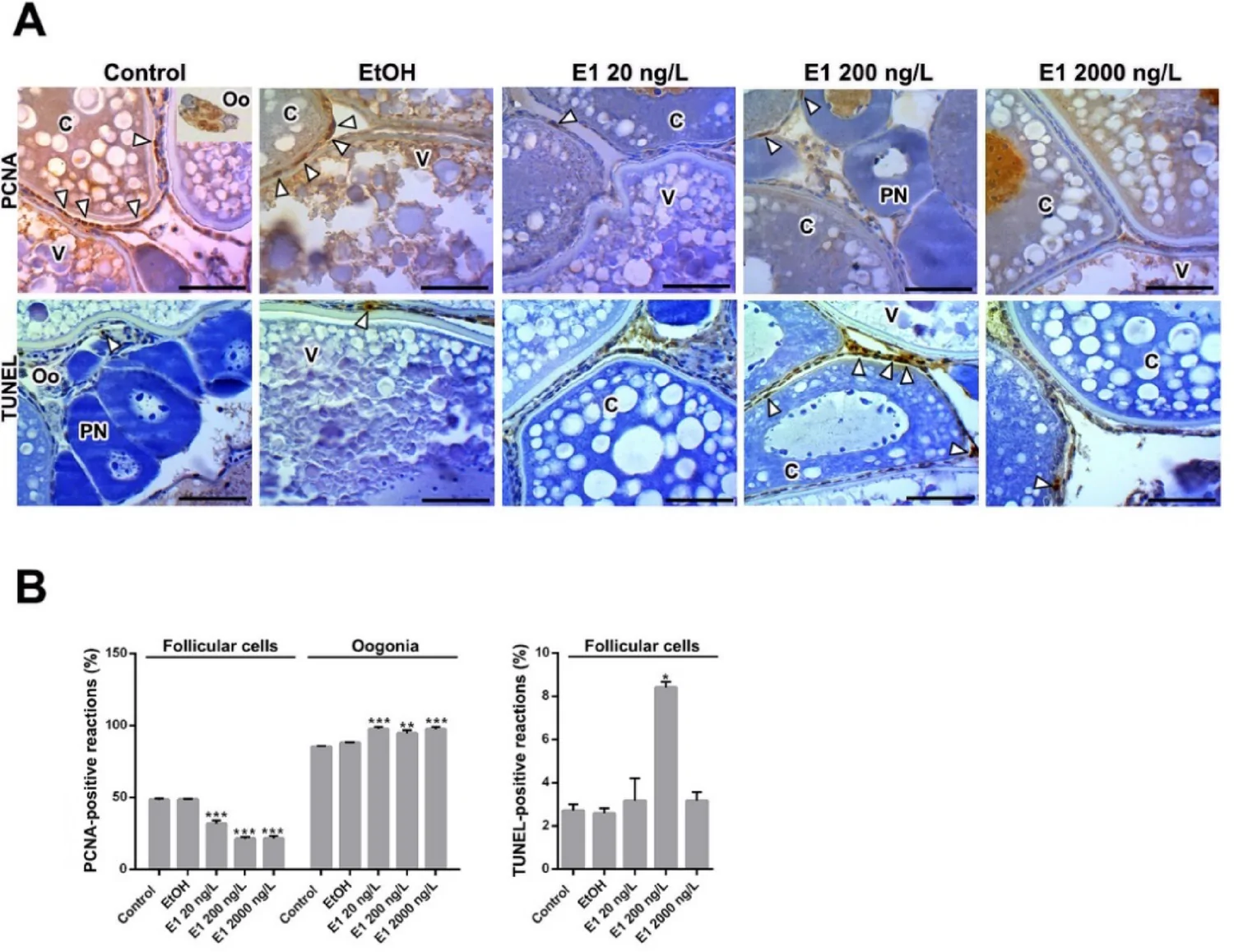
**A** Immunostaining for PCNA and TUNEL reaction in Danio *rerio* ovaries exposed to E1 for 49 days. Sections were counterstained with hematoxylin. Note labelling for PCNA and TUNEL in follicular cells (white arrowhead) in all groups. Bar scale = 50 µm. **B** Quantification of PCNA and TUNEL labeling in zebrafish ovaries: note proliferation (PCNA-positive rection) of oogonia and follicular cells, and follicular cell apoptosis (TUNEL reaction). Data are expressed as mean ± SEM from six fish per group (*p < 0.05, ** p < 0.01, *** p < 0.001)

The PCNA and TUNEL assays showed stained follicular cells in all experimental groups (Fig. 4A). Oogonia and oocytes were not stained for TUNEL, and PCNA-positive staining in oogonia is not shown. Quantitative analyses (Fig. 4B) indicated a significant increase in cell proliferation (PCNA-positive cells) in oogonia from the groups exposed to E1 (*p* < 0.01), whereas proliferation in follicular cells was markedly reduced compared to the control (*p* < 0.001). Additionally, cell death (TUNEL-positive cells) in follicular cells was significantly elevated in the E1 200 ng/L group relative to the control (*p* < 0.05).

### Immunohistochemistry for IGF1. IGF2, and IGF1R

**Fig. 5:**
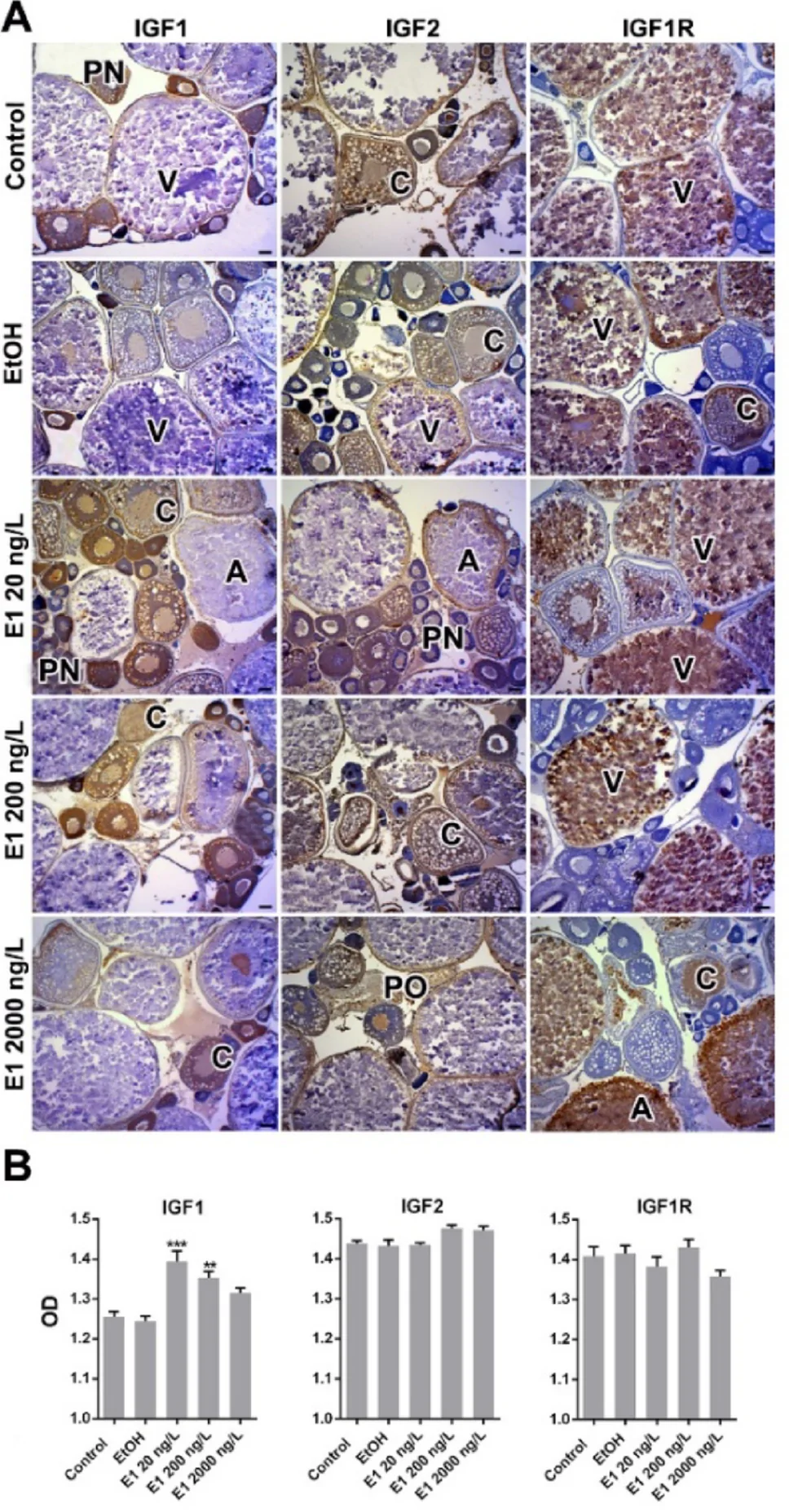
**A** Immunostaining for IGF1, IGF2 and IGF1R in Danio *rerio* ovaries exposed to E1 for 49 days. Sections were counterstained with hematoxylin. Note immunoreaction in perinucleolar oocytes (PN), oocytes with cortical alveoli (**C**), vitellogenic oocytes (V), postovulatory follicles (PO) and atretic follicles (**A**). Bars scale = 50 µm. **B** Quantification of the immunoreactions for optical densitometry (OD) showing increased IGF1 immunolabeling in E1 20 and 200 ng/L groups, whereas no significant differences were observed for IGF2 and IGF1R. Data are expressed as mean ± SEM from six fish per group (*p < 0.05, ** p < 0.01, *** p < 0.001)

Positive immunostainings for IGF1 and for IGF2 were detected in the cytoplasm of oocytes and follicular cells at all developmental stages, as well as in postovulatory follicles and atretic follicles (Fig. 5A). IGF1R staining was observed in the oocytes and follicle cells at cortical alveoli, vitellogenic, postovulatory, and atretic follicles (Fig. 5A). Quantitative analysis revealed that IGF1 immunolabeling significantly increased in the 20 and 200 ng/L E1 groups compared to the control (*p* < 0.01; Fig. 5B). Despite a trend towards increased IGF2 expression in the E1 200 and 2000 ng/L groups, and reduced IGF1R expression in the E1 20 and 2000 ng/L groups, no significant differences were observed in IGF2 and IGF1R among the treated groups (*p* > 0.05).

### Immunohistochemistry for iNOS and Nitrite Production

**Fig. 6:**
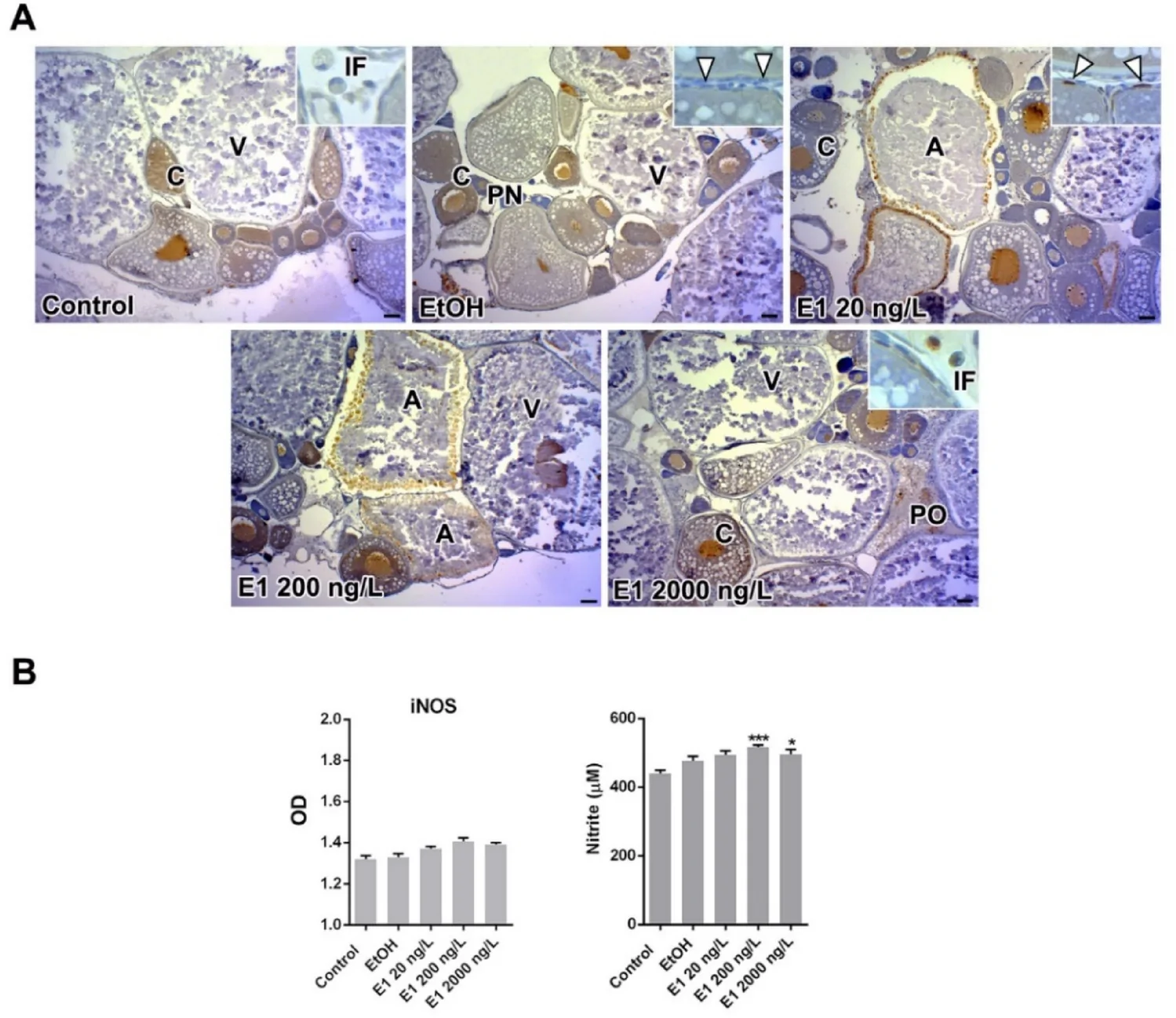
**A** Immunohistostaining for iNOS in ovaries of *Danio rerio* exposed to E1 for 49 days. Sections were counterstained with hematoxylin. Note nuclear immunoreaction in different follicular developmental stages: perinucleolar (PN), cortical alveoli (**C**) and vitellogenic follicles. Note also immunoreaction for iNOS in follicular cells (white arrowhead) in vitellogenic (V), postovulatory follicles (POF) and atretic follicle (**A**). Cells in inflammatory infiltrate (IF) were also marked. Bar scale = 50 µm. **B** Quantification of the immunoreactions for iNOS and nitrite production in zebrafish ovaries. Note not significant reactions for iNOS and significant increase for nitrite production at E1 200 and 2000 ng/L groups. Data are expressed as mean ± SEM from six fish per group (*p < 0.05, ** p < 0.01, *** p < 0.001)

Immunohistochemical analysis revealed iNOS labelling in all developmental phases of the ovarian follicle, as well as in postovulatory and atretic follicles in all E1-exposed groups (Fig. 6A). Notably, follicular cells from E1-exposed fish exhibited more intense labeling, while staining on the inflammatory infiltrate was observed only in E1-exposed fish. Although the optical density of iNOS labeling in the ovaries did not differ significantly between treatments, a trend toward increased expression was evident in the E1-exposed fish compared to the control, with the highest levels observed in the E1 200 ng/L group (Fig. 6B). In addition, nitrite production–a marker of nitric oxide–was significantly elevated in the E1 200 ng/L and 2000 ng/L groups (*p* < 0.05; Fig. 6).

### Egg Production and Fertilization Rate

**Fig. 7:**
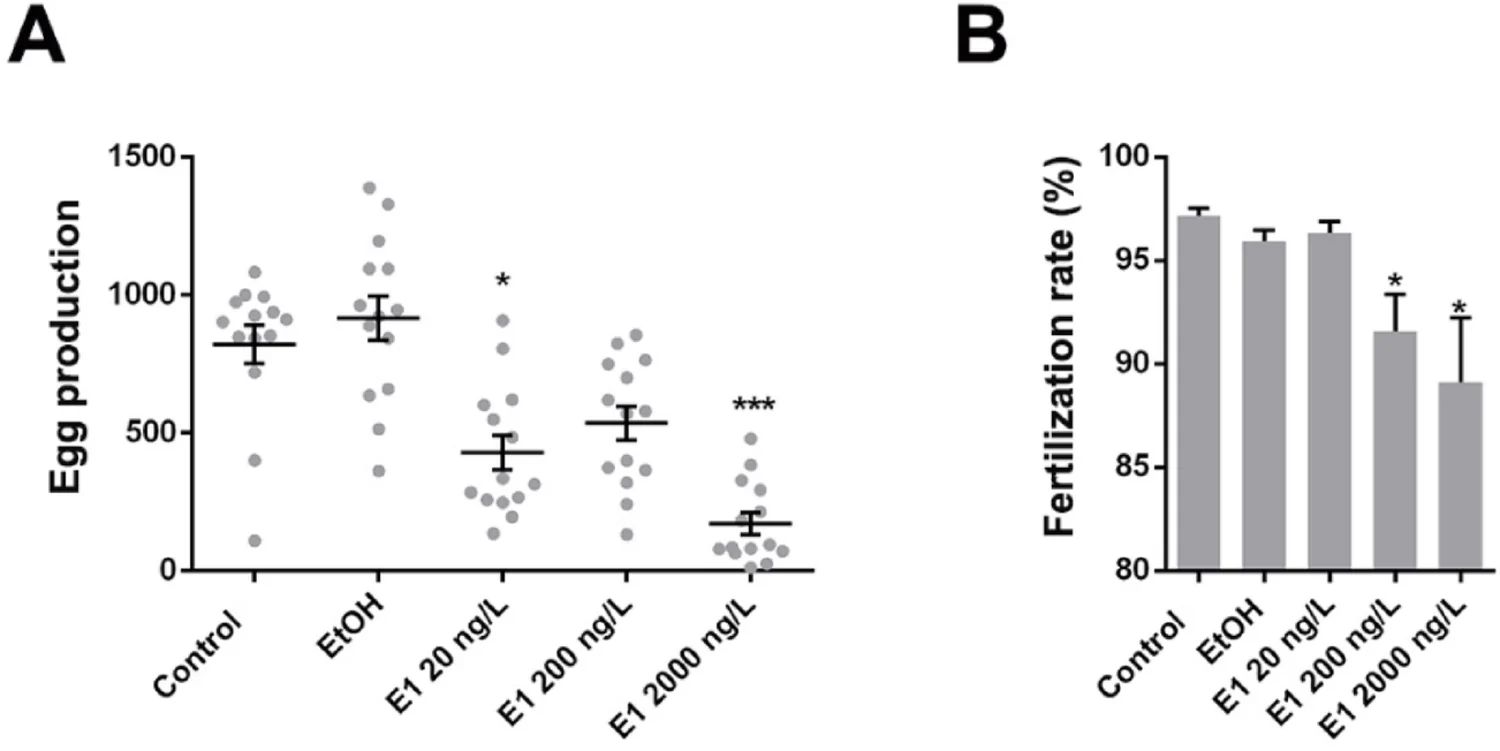
Egg production (**A**) and fertilization rate (**B**) in zebrafish *Danio rerio* in the groups: control, ethanol (EtOH) and exposed to different concentrations of estrone: E1 20, 200 and 2000 ng/L for 49 days. Note significantly reduced egg production at E1 20 and 2000 ng/L groups and reduced fertilization rate at E1 200 and 2000 ng/L groups. Data represent mean ± SEM from six females per group (*p < 0.05, ** p < 0.01, *** p < 0.001)

Egg production, measured as the number of eggs spawned, was significantly reduced in the E1 20 ng/L and 2000 ng/L groups (*p* < 0.05; Fig. 7A). Additionally, the fertilization rate, defined as the percentage of fertilized eggs, was markedly lower in the E1 200 ng/L and 2000 ng/L groups (*p* < 0.05; Fig. 7B).

## Discussion

The widespread presence of estrogens in aquatic environments is a significant public concern, as even low concentrations can threaten environmental integrity, animal health, and human well-being (Silva et al. 2025). In the present study, E1 exposure increased E2 levels in ovaries, reduced follicular cell proliferation, increased IGF1 expression and oxidative stress, and impaired ovarian germinal epithelium organization in zebrafish. These effects were associated with increased follicular atresia, spawning, and fertilization rates, demonstrating that environmentally relevant concentrations of E1 compromise zebrafish reproductive physiology.

While the increase in the GSI typically reflects gonadal maturation under normal conditions, our findings indicate that GSI and spawning were both reduced in the 20 ng/L E1-exposed group. Notably, GSI remained unchanged in the E1 2000 ng/L group probably due to low number of spawned eggs. This is because GSI alone may not provide a reliable indication of gonadal maturation or alterations in gametogenesis, particularly under conditions of environmental stress and impact (Madureira et al. 2011; Ribeiro et al. 2021). Additionally, E1 exposure did not affect the K factor, indicating that the nutritional status and overall health of the zebrafish remained stable throughout the experiment.

Alterations in the proportions of germ and somatic cells within the gonads, as observed in this study, are well-documented effects in fish exposed to estrogenic compounds (Prado et al. 2014; Weber et al. 2019; Moreira et al. 2023). These changes are largely attributed to disruptions in estrogen and androgen levels, which directly regulate the proliferation, survival, and death of germ cells (Weber et al. 2019; Paschoalini et al. 2021; Ribeiro et al. 2021). In this study, the observed increase in gonadal E2 concentrations following exposure to E1 at 20 and 200 ng/L may be related to the conversion of E1 to E2 by 17β-hydroxysteroid dehydrogenase (17β-HSD) in combination with aromatase (CYP19), both key enzymes in the final steps of estrogen biosynthesis (Tokarz et al. 2015). Interestingly, this elevation in E2 was not detected in females exposed to 2000 ng/L of E1, likely reflecting a non-monotonic dose–response relationship for E2 levels in zebrafish subjected to increasing E1 concentrations—a pattern frequently reported following exposure to EDCs (Lagarde et al. 2015; Wu et al. 2021).

Both E2 and 11-KT are essential for ovarian development and maturation in teleost fish (Lubzens et al. 2010; Wang et al. 2020). Notably, 11-KT is recognized as a potent inducer of primary oocyte growth during the previtellogenic phase (Wang et al. 2020; Monson et al. 2017, 2024). In this study, ovarian levels of 11-KT did not change significantly following E1 exposure, indicating that previtellogenic oocyte growth was not affected by E1. Conversely, the elevation of E2 levels was accompanied by a marked reduction in 11-KT in zebrafish males exposed to E1, resulting in decreased sperm production and semen quality (Ribeiro et al. 2023). Collectively, these findings demonstrate that exposure to environmentally concentrations of E1 disrupts reproductive physiology in both male and female fish.

Although increased levels of E2 did not significantly alter the proportion and size of vitellogenic follicles, they were sufficient to stimulate oogonial proliferation, as also demonstrated in previous studies (Higashino et al. 2002; Lubzens et al. 2010). Consistently, E2 influences different phases of ovarian physiology, promoting both oogonial proliferation and vitellogenesis; conversely a reduction in E2 levels during the steroidogenic shift is associated with the onset of final oocyte maturation (Lubzens et al. 2017). In addition to E2, both IGF1 and IGF2 are recognized for their proliferative effects, promoting progression of oogenesis in fish (Reinecke 2010; Moreira et al. 2020). Previous studies have shown that hepatic and gonadal expression of these peptides can be modulated by estrogenic EDCs (Reinecke 2010; Prado et al. 2014). In the present study, increased IGF1 expression was detected in females exposed to E1, suggesting that IGF1 may also contribute to the enhanced proliferation of oogonia, given its mitogenic activity. Furthermore, IGF1 promotes granulosa cells differentiation and is required for follicle growth induced by follicle-stimulating hormone (FSH) (Zhou et al. 2013).

In this study, E1 exposure in female zebrafish increased the proportion of atretic follicles, a pattern consistent with the effects of other environmental estrogens, such as ethinyl estradiol, nonylphenol, bisphenol A, and diethylhexylphthalate, all of which are known to disrupt the production of gonadotropins and sex steroids (Mandich et al. 2007; Kaptaner and Ünal 2011; Ye et al. 2014). The deleterious ovarian effects observed may be mediated by alterations in follicular cells, whose proportion was reduced due to decreased proliferation and increased cell death, particularly in the E1 200 ng/L group. Since the production of E2 depends on follicular cells, a reduction in these cells likely contributes to impaired follicular development and oocyte maturation, followed by increased follicular atresia. Indeed, exposure to environmental contaminants that affect the number and function of follicular cells can negatively impact the development and maturation of ovarian follicles, as well as ovulation (Lubzens et al. 2010; Corriero et al. 2021). In this regard, the reduction in follicular cells and increase in follicular atresia detected in E1-exposed groups in this study were accompanied by decreased egg production and fertilization rates. In a previous study, males exposed to E1 at the same concentrations used in this study exhibited negative effects on important sperm parameters, particularly progressive motility and beat-cross frequency in the E1 20 and 2000 ng/L groups (Ribeiro et al. 2023). This data may justify the reduced fertilization rate observed in the E1 2000 ng/L group in the present study. Interestingly, a study conducted with fathead minnow, *Pimephales promelas*, exposed to E1 at concentrations of 10, 50, and 100 ng/L demonstrated a non-monotonic effect on fertilization rates (Dammann et al. 2011). This pattern differed from that observed in the present study, suggesting species-specific responses to E1 exposure. This finding highlights the complex effects of E1 and other estrogens on fish gametogenesis and reproduction, as observed in other studies (Schäfers et al. 2007; Dammann et al. 2011; Gárriz et al. 2015; Lei et al. 2023).

The role of NO is well documented in the regulation of mammalian reproduction, but few studies have addressed its function in other vertebrates. In fish ovaries, NO stimulates folliculogenesis, vitellogenesis, and steroidogenesis, depending on the status of follicular development and NO concentration (Singh and Lal 2017). In the present study, the increase in nitrite production in the E1 200 and 2000 ng/L groups was associated with a greater incidence of atretic follicles. Interestingly, this rise in NO was not accompanied by increased iNOS expression, indicating that other NOS isoforms may have been affected by estrogenic stimulation. In this regard, ovarian eNOS is frequently increased in response to cytokine signaling or oxidative stress in mice ovary, especially under pathological conditions (Shen et al. 2012). In the fish *Heteropneustes fossilis,* increased NO production disrupted germ vesicle breakdown and meiosis progression in oocytes (Tripathi and Krishna 2008). In pigs, oxidative stress triggers granulosa cell apoptosis via the MAPK signaling pathway during follicular atresia (Zhao et al. 2025). Our results suggest that oxidative stress induced by NO may impair follicular cells proliferation, as evidenced by their reduced proportion and increased apoptosis (TUNEL reaction), thus increasing follicular atresia. Indeed, cell proliferation and apoptosis ensure an adequate number of germ cells during gonadal development and the quality of the gametes production (Thomé et al. 2012; Melo et al. 2015; Ribeiro et al. 2017).

## Conclusion

Overall, the findings of the present study demonstrate that E1 exerts deleterious effects on ovarian development, compromising oocyte maturation, spawning, and fertilization in zebrafish. The imbalance of sex steroid production, accompanied by effects on the IGF signaling pathway and nitric oxide production, contributes negatively to the progression of oogenesis, spawning, and oocyte quality in the exposed groups. Based on the observed effects, our results indicate that E1, even in environmentally relevant concentrations, compromises fish reproductive physiology. Monitoring programs must be implemented to control environmental estrogens and conserve aquatic ecosystems to ensure animal health and human well-being.

## Data Availability

The data supporting this study may be made available by the author upon request.
